# The Many Lives of Staph: Staphylococcal Scalded Skin Syndrome in Two Brothers Secondary to Staphylococcus aureus-Associated Ecthyma Gangrenosum

**DOI:** 10.7759/cureus.85122

**Published:** 2025-05-31

**Authors:** Madeline Alizadeh, Laurene Dampare, Saskia Groenewald

**Affiliations:** 1 Institute for Genome Sciences, University of Maryland School of Medicine, Baltimore, USA; 2 Family Medicine, University of Maryland School of Medicine, Baltimore, USA; 3 Pediatrics, Baltimore Washington Medical Center, Glen Burnie, USA

**Keywords:** ecthyma gangrenosum (eg), pediatric infection, skin infections, staph-aureus, staphylococcal scalded skin syndrome

## Abstract

*Staphylococcal* scalded skin syndrome (SSSS) is a rare, infectious entity whereby the systemic distribution of *Staphylococcal* toxins results in the development of multiple symptoms, the most notable of which is a desquamative rash. Ecthyma gangrenosum, a skin infection most frequently occurring in immunocompromised individuals, is similarly uncommon. Herein, we present the case of two young siblings presenting with SSSS following the development of ecthyma gangrenosum. Prompt treatment of the *Staphylococcal* infection led to resolution of symptoms and recovery. Our case highlights the need to better elucidate the underlying factors driving variation in clinical response to *Staphylococcal aureus* infections.

## Introduction

*Staphylococcal* scalded skin syndrome (SSSS) is a systemic response to *Staphylococcal* toxins. The name of the condition originates from the burned or scalded appearance that the skin develops as the disease progresses. In addition to a blistering, desquamative rash, patients frequently develop constitutional symptoms such as fever, irritability, and malaise [1,2]. The disease is observed most frequently in otherwise healthy children, typically following upper respiratory tract, ear, or eye infections, but is much less likely to occur beyond the age of two; this is hypothesized to be due to antibody development and rising titers to the exfoliative *Staphylococcal* toxins [2]. There is a slight male predominance, with an estimated incidence of 0.09 to 0.56 cases per million [3,4]. Ecthyma gangrenosum, in contrast, represents a disseminated skin infection, with systemic spread of bacteria from multiple lesions, rarely seen in immunocompetent children [5,6]. It is typically observed in immunocompromised or, less often, healthy adults. While *Pseudomonas* is the most common causative agent, ecthyma-like lesions due to *Staphylococcal aureus* infection may be observed as the bacterial source [6,7]. Our case describes the previously unreported development of SSSS secondary to the development of rare, ecthyma-like lesions in two previously healthy young boys. We highlight that although atypical, skin infections caused by *S. aureus *may involve strains that can cause SSSS, and further work characterizing host factors responsible for variation in response to* S. aureus* infection is required.

## Case presentation

A 2.5-year-old (older brother, OB) previously healthy male presented to our emergency department (ED) with facial, neck, upper truncal, and axillary erythema and swelling; perioral peeling; and a lesion on his medial right thigh. His mother reported he was in his usual state of health three and a half days prior when she and her husband dropped the patient and his brother off for the weekend with family before leaving for a trip. Two days later, his parents were told he developed congestion and a “high fever” for which he was given acetaminophen. At this time, he had also developed mild facial swelling. One day later, the swelling extended from his face down to the neck, upper trunk, and axilla, accompanied by diffuse erythema, some perioral peeling, and a dark lesion on his right medial thigh, prompting them to present to our ED. His 1.5-year-old younger brother (YB) also had two similar lesions on his lower extremities and was febrile at that time.

In the two to three hours following admission (hospital day 1 (HD1), OB), swelling and redness significantly worsened, but upper respiratory infection (URI) symptoms remained stable. The parents denied diarrhea or vomiting, but the OB complained of significant body aches. His only new medication exposure was fexofenadine, taken three days prior, with no other recent medication or supplement use prior to the start of symptoms. The URI PCR panel, which included *Bordetella pertussis, Chlamydia pneumoniae, Mycoplasma pneumoniae*, multiple types of coronavirus (including SARS-CoV-2), influenza, human metapneumovirus, parainfluenza, rhinovirus/enterovirus, and RSV, was positive for RSV alone. He had no past medical or surgical history and no known drug allergies. All vaccines and immunizations were up to date, and there was no relevant family history of autoimmune, dermatologic, or atopic disease. Just prior to the parent's trip, however, their father had gotten a new tattoo on his calf, and broken out in what looked like “pimples or boils” in the area surrounding the tattoo.

**Figure 1:**
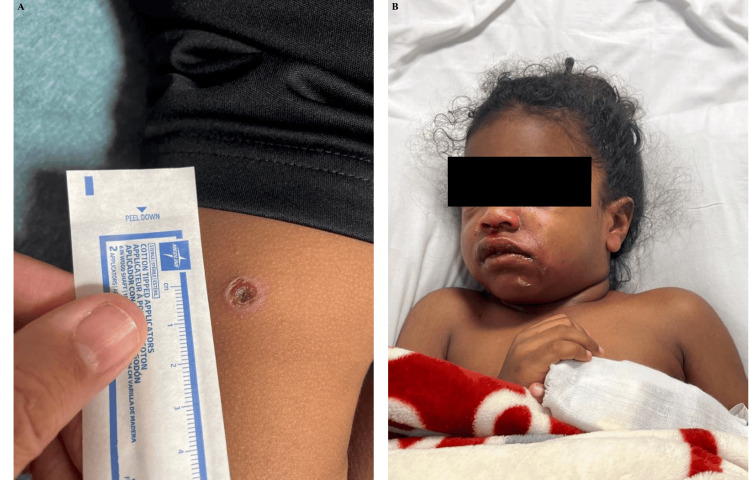
Images of OB on HD1. (A) Presentation of ecthyma on HD1. (B) Facial swelling, erythema, and desquamation at presentation. OB, older brother; HD, hospital day

On exam, OB was febrile (39.3), irritable, and difficult to console but hemodynamically stable with elevated pressures (138 bpm, within normal for age, and 144/107) and without stridor or signs of respiratory distress (respiration rate 32, SpO_2_ 100%, within normal for age). He had mild congestion, copious rhinorrhea, and watery conjunctival discharge. Mucous membranes were moist, and the oropharynx was clear, with no posterior oropharyngeal erythema. Mild coarse breath sounds were present on expiration. A patchy, erythematous rash extended over the facial, auricular, neck, axillary, and upper truncal surfaces to the level of the axilla (Figure 1). Significant periorbital, perioral (with lip involvement), auricular (R>L) neck, and axillary edema were present, with associated tenderness limiting movement, especially of the arms. Appreciable desquamation of perioral skin was observed. Cervical, mandibular, and pre/post-auricular lymph nodes could not be palpated or appreciated due to significant regional swelling. Scrotal peeling and swelling were present, and a necrotic-appearing lesion on the medial right thigh thought to be an ecthyma-like lesion was observed (Figure 1). He demonstrated no muscle weakness, normal reflexes, and benign cardiovascular and abdominal examinations, with all other findings being normal. CBC, CRP, and ESR were all within normal limits (WBC = 6.7 x 10^3 ^WBC/mm^3^; Table 1), though low bicarbonate (17 mmol/L) and an elevated anion gap (18) were observed (Table 2). Urinalysis revealed ketonuria (60 mg/dL) and proteinuria (50 mg/dL). Blood cultures and nasopharyngeal swabs were negative for *S. aureus*.

**Table 1:** CBC of OB throughout the hospital stay. WBC, white blood cell; MCH, mean corpuscular hemoglobin; MCHC, mean corpuscular hemoglobin concentration; MCV, mean corpuscular volume; MPV, mean platelet volume; RBC, red blood cell; RDW, red cell distribution width; HCT, hematocrit; OB, older brother; HD, hospital day

	Latest Reference Range and Units	HD1	HD2	HD4
WBC	4.9 - 13.0 K/mcL	6.7	7.1	9.4
Hemoglobin	11.5 - 15.0 g/dL	13.0	12.8	12.6
HCT	33.0 - 42.0%	37.6	37.4	36.6
Platelets	150 - 400 K/mcL	338	304	346
Neutros Abs	1.90 - 8.70 K/mcL	3.96	1.06	2.99
Neutros %	12 - 35%	60 (H)	15	32
Lymphocytes Absolute	2.00 - 12.00 10*9/L	2.05	5.11	5.13
Lymphs %	35 - 75%	31 (L)	67	55
Monocytes Absolute	0.10 - 2.80 10*9/L	0.60	0.78	0.37
Monos %	0 - 9%	9	11	4
Basophil Abs	0.00 - 0.20 10*9/L	0.03	0.00	0.03
Basophil %	0 - 2%	0	0	0
Eosinophils Absolute	0.00 - 0.45 10*9/L	0.01	0.14	0.80 (H)
Eos %	0 - 6%	0	2	9 (H)
Nucleated RBC Abs	0.00 - 0.00	0.00	0.00	0.00
Nucleated RBC%	0 - 0%	0	0	0
Hypochromasia			1+	
MCH	25.0 - 30.0 pg	27.2	27.1	27.2
MCHC	30.0 - 36.5 g/dL	34.6	34.2	34.4
MCV	76.0 - 86.0 fL	78.7	79.2	78.9
MPV	8.8 - 11.5 fL	8.5 (L)	8.5 (L)	8.6 (L)
RBC	3.90 - 5.50 M/mcL	4.78	4.72	4.64
RDW	11.5 - 14.5%	12.9	13.2	12.8
Immature Grans (Abs)	0.00 - 0.10 10*9/L	0.02		0.03

**Table 2:** Complete metabolic panel and CRP of OB throughout the hospital stay. BUN, blood urea nitrogen; AST, aspartate aminotransferase; ALT, alanine aminotransferase; OB, older brother; HD, hospital day

	Latest Reference Range and Units	HD1	HD2	HD3	HD4
Sodium	136 - 145 mmol/L	137	137	140	140
Potassium	3.5 - 5.1 mmol/L	4.1	3.9	4.5	4.5
Chloride	98 - 107 mmol/L	102	109 (H)	110 (H)	106
CO_2_ Total	22 - 29 mmol/L	17 (L)	20 (L)	20 (L)	23
Anion Gap	2 - 11	18 (H)	9	9	11
BUN	5 - 18 mg/dL	11	5	<4 (L)	4 (L)
Creatinine	0.3- 0.7 mg/dL	0.35	0.29	0.29	0.44
BUN/Creatinine Ratio		31	17	<14	9
Glucose, Blood	74 - 109 mg/dL	92	95	99	81
Calcium	8.8 - 10.8 mg/dL	9.7	9.0	8.7 (L)	9.1
Total Protein	6.4 - 8.3 g/dL	7.0	5.6 (L)	4.9 (L)	6.1 (L)
Albumin	3.5 - 5.2 g/dL	4.5	3.6	3.2 (L)	3.8
AST	≤50 U/L	46	39	35	34
ALT	≤50 U/L	23	20	18	21
Bilirubin Total	≤1.20 mg/dL	0.58	0.26	0.20	0.22
Alkaline Phosphatase	142 - 335 U/L	245	183	162	195
CRP	≤5 mg/L	0.6	2		<3

**Figure 2:**
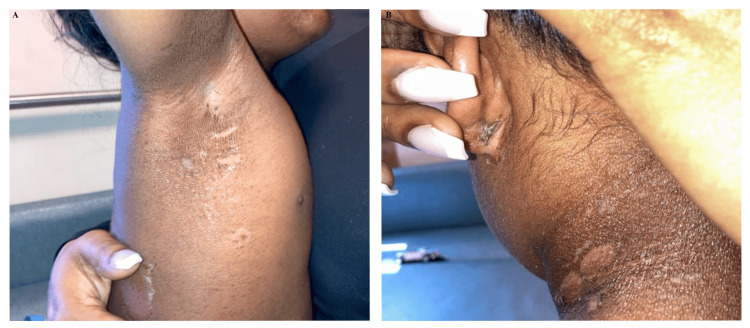
Progression of OB's rash on HD4. (A) Axillary desquamation and (B) postauricular and neck desquamation following resolution of swelling on HD4. OB, older brother; HD, hospital day

Given the high degree of suspicion for SSSS, the clinical diagnosis was made and broad-coverage antibiotic treatment (clindamycin 13 mg/kg and vancomycin 15 mg/kg, IV q6) was immediately initiated. OB’s swelling and rash stabilized, and by HD2 he was less distressed and irritable, though he developed swelling in his right hand; vancomycin was discontinued at this time and replaced by oxacillin (25 mg/kg, IV q6). By HD3, the swelling had resolved, and he showed significant improvement in effect, activity, pain, rash redness, and edema, along with normalization of previously abnormal laboratory values. Oxacillin was discontinued at this time. When the swelling had significantly decreased on HD3, spreading desquamation of the affected surfaces was noted, initially with 5-8% total body surface area involvement. By HD4, swelling had improved by greater than 75%, and desquamation involved 8-10% of his body surface area, including perioral, right auricular, neck, scrotal, and bilateral axillary surfaces, but wounds remained dry, without weeping or exudate (Figure 2); he was discharged with six days of oral cephalexin (25mg/kg QID) for a total of 10 days of antibiotic treatment. Swabs from the underside of his lesion were positive on culture for methicillin-sensitive *S. aureus* (HD4; Table 3).

**Table 3:** Sensitivity profile of MSSA-positive swabs for OB. MIC, minimum inhibitory concentration; Methicillin-sensitive *Staphylococcus aureus*;* *OB, older brother

Antibiotic	Interpretation	MIC
Cefazolin	Sensitive	
Ciprofloxacin	Sensitive	≤1
Clindamycin	Resistant	≤0.25
Daptomycin	Sensitive	0.5
Erythromycin	Resistant	>4
Gentamicin	Sensitive	≤1
Levofloxacin	Sensitive	≤0.5
Linezolid	Sensitive	1
Oxacillin	Sensitive	≤0.25
Rifampin	Sensitive	≤1
Tetracycline	Sensitive	≤2
Trimethoprim/Sulfamethoxazole	Sensitive	≤0.5/9.5
Vancomycin	Sensitive	1

**Figure 3:**
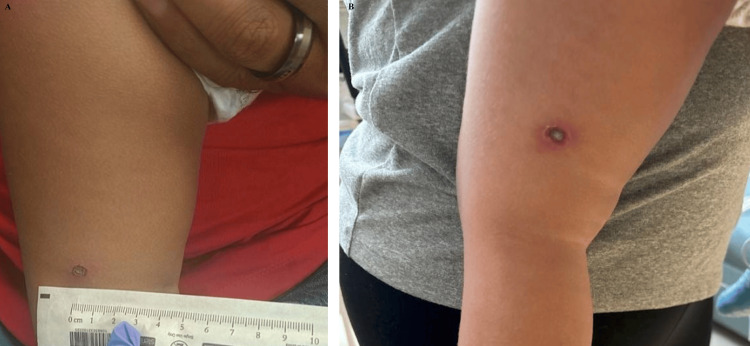
Presentation and progression of one ecthyma lesion on YB. Ecthyma on the left lower extremity of YB on his (A) HD1 and (B) HD2. YB, younger brother; HD, hospital day

**Figure 4:**
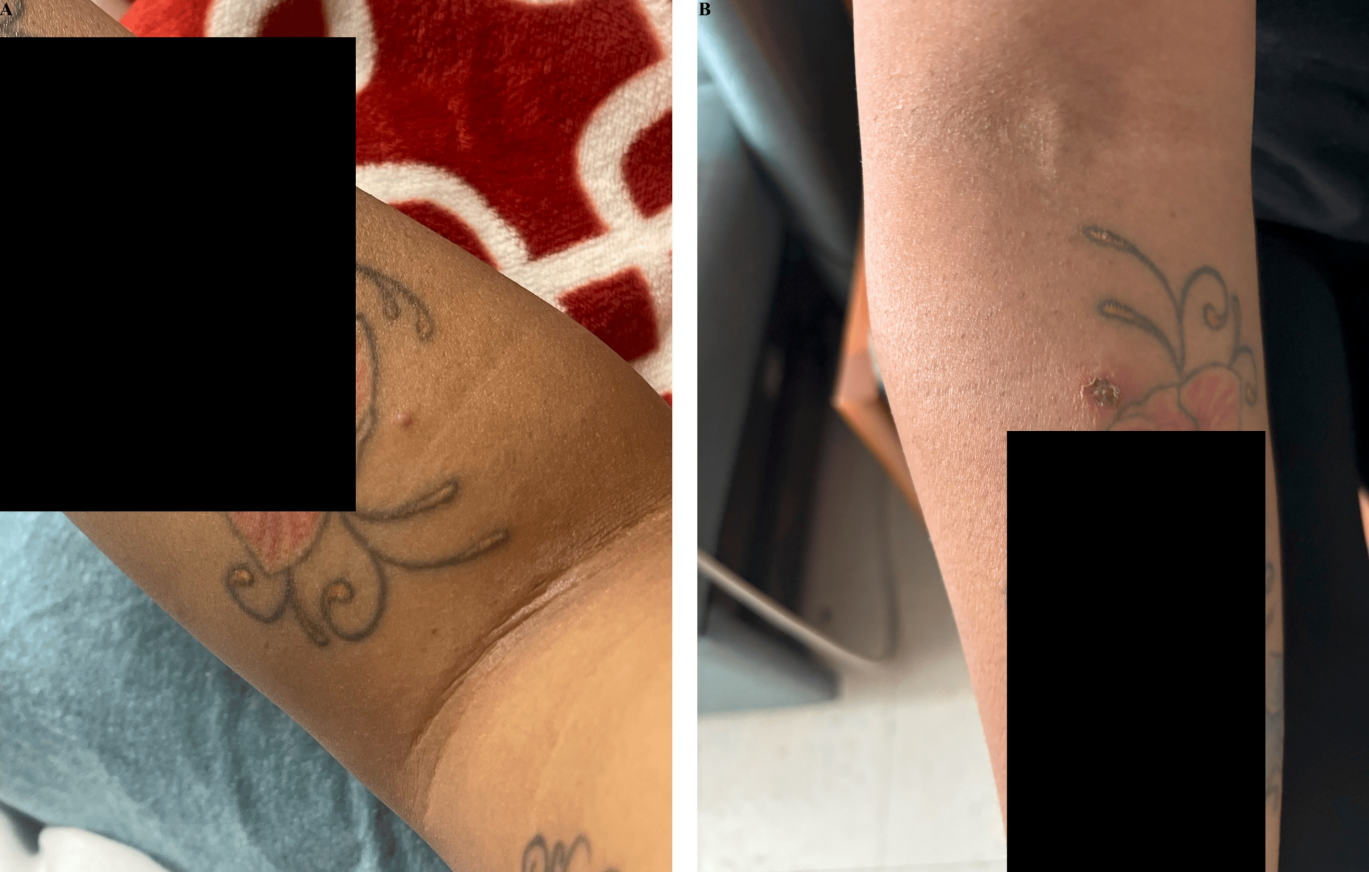
Progression of pustule to ecthyma. A representative pustule on the boy's mother, observed two to three days prior to OB's hospitalization, which ulcerated and developed into (B) an ecthymatous lesion on OB's HD4 (i.e., YB's HD2). YB, younger brother; OB, older brother; HD, hospital day

During this time (OB, HD3), YB was noted to have additional lesions on his trunk and leg, which had initially presented as pustules, similar to those surrounding their father’s tattoo (Figure 3), and worsening symptoms including the development of facial swelling similar to OB’s initial presentation, prompting his parents to bring him to the ED (OB, HD4) where he was admitted (YB, HD1). On exam, he was febrile (40.5) but was normotensive (116/66) and without tachycardia (126 bpm, within normal for age), tachypnea, and stridor or signs of respiratory distress (respiratory rate 30, SpO_2_ 96%, within normal for age). The same URI panel described above was run and was positive for RSV alone. Mild facial, neck, and upper truncal/axillary swelling and redness were present, though not as extensive as those observed in OB, and clindamycin and cefazolin (13 mg/kg and 25 mg/kg, respectively, IV q6) were initiated. Swabs of his open lesions were also positive for *S. aureus *(sensitivity profile was not provided). On YB's HD2, their mother had a similar ulcerated-appearing lesion develop, which similarly started as a pustule (Figure 4) but developed no other symptoms. By YB's HD4, the swelling was gone, he was afebrile, and the axillary and inguinal rash had nearly resolved with the improvement of his facial rash; he was similarly discharged with six days of oral cephalexin (25 mg/kg QID) to be taken at home. One week following YB’s discharge, both boys were back to their normal state of health.

## Discussion

Our patients were previously healthy almost 2- and 3-year-old males with no past medical history who presented with diffuse facial, cervical, and upper truncal redness; swelling; and desquamative rash secondary to SSSS, following the development of ecthyma-like lesions. SSSS classically presents with erythema, fever, and the development of large fluid-filled bullae, which may rupture to slight pressure (positive Nikolsky sign), leaving behind desquamated patches of skin [1]. Most frequently occurring in younger children [1], SSSS typically affects the groin, axillae, and face, without mucosal involvement [8], and labwork is often unremarkable [4]. It is caused by the release of exfoliative toxins from specific strains of *S. aureus*, which interact with desmoglein-1 and behave as atypical serine proteases [9]. As a result, desquamation associated with SSSS tends to occur more superficially [9], and histology demonstrates superficial intra-epidermal cleavage occurring below the stratum corneum [8]. Diagnosis is typically clinical and involves ruling out other potential causes (e.g., Stevens-Johnson syndrome or toxic epidermal necrolysis) though it can be confirmed with skin biopsy in cases where diagnosis is unclear [6]. Because there was a lack of mucous membrane involvement combined with rapid response to antibiotics, as well as an identified nidus of infection, in our patients the diagnosis was clear and we opted not to pursue a biopsy. Sensitive and specific serum biomarkers that are clinically available do not exist and would greatly help in cases of uncertainty.

In contrast, ecthyma gangrenosum is a rare dermatologic condition. Lesions that begin as painless macules become erythematous nodules or pustules, eventually developing into erythematous ulcers with a central area of necrosis becoming gray-black eschars [10], precisely as they did in our patients. Lesions may be caused by *S. aureus;* however, *Pseudomonas aeruginosa* is the most common causative organism, particularly in immunocompromised individuals; nonetheless, specific bacterial etiology has little bearing on clinical presentation and management [7]. It can occur in those with or (far less commonly) without immunocompromise, and with or without associated septicemia [11], though *Pseudomonas* and rarer organisms may be the likelier culprit in the immunosuppressed population [12]. It is distinguished from ecthyma by features indicating systemic dissemination, such as septicemia/bacteremia; however, the two can still be difficult to separate, with conflicting opinions in the literature regarding whether they represent a single or two distinct clinical entities [7].

Outbreaks of SSSS may occur in settings such as daycares, indicating the potentially contagious nature of causative strains [13]. Genetic variability in the site of action of the exfoliative toxin has also been posited to play a role in susceptibility to SSSS [14], which has been reported following a range of localized infections, including conjunctivitis, pneumonia, septic arthritis, pyomyositis [8], and even skin lesions [15]. Thus, it is not unreasonable that ecthyma gangrenosum caused by an exfoliative toxin-producing strain of *S. aureus* may have provided a nidus for bacterial toxin introduction in both boys. Interestingly, even though both parents had skin findings consistent with *S. aureus* infection, in the form of identical ecthyma-like lesions in their mother and several pustules without progression to ecthyma in their father, neither developed SSSS. SSSS is rare in adults and carries up to a 60% mortality rate in the non-pediatric population [9]. Ecthyma gangrenosum, in contrast, occurs in immunocompromised individuals in a majority of cases. In healthy individuals who develop it, immunodeficiency or other risk factors (particularly hematologic malignancy) are frequently, though not always, identified later [16]. The development of ecthymatous lesions in three related members of the household (a parent and two children) without a similar lesion developing in the father perhaps indicates an underlying genetic susceptibility. Beyond immunodeficiency, genetic variability in host response to infectious diseases is common [17]. Thus, it is not unreasonable that while some strains of *S. aureus* may be causative of certain forms of infection, genetic differences are at the basis of susceptibility and/or clinical presentation of ecthyma gangrenosum as well.

Strengths of our case workup include our ability to care for and observe multiple members of the family, specifically the two siblings affected by SSSS. Additionally, we were able to watch the patients' progression over several days and received an updated report from their mother a few weeks following discharge. Finally, we were able to do a thorough clinical work-up, including multiple sets of cultures from various sample types (i.e.,* *serum, lesion, and nasal swabs). There were, however, limitations; this included our inability to strain-type *S. aureus *detected on swab samples and perform further experiments to characterize the microbes involved. We were also unable to do more extensive, experimental (e.g.,* *exploratory genetic) follow-up to identify potential underlying causes for the clinical presentation we observed.

## Conclusions

In summary, we present the case of two young brothers who developed SSSS secondary to ecthyma gangrenosum. Ecthyma gangrenosum represents systemic infection from ecthyma-like lesions, which in our case, led to SSSS. Given the potential for rapid progression of these infections and associated mortality in untreated individuals, prompt recognition and treatment initiation are of the utmost importance. Our case highlights the complexity of *S. aureus *infection presentation and the need for an improved understanding of what factors drive clinical presentation. Due to the rarity of these conditions, accurately identifying host factors is difficult but will likely improve over time as high-throughput methods are increasingly employed to expand precision medicine.
